# Effect of a lactate‐guided conditioning program on heart rate variability obtained using 24-Holter electrocardiography in Beagle dogs

**DOI:** 10.1371/journal.pone.0233264

**Published:** 2020-06-01

**Authors:** Alejandro Z. Restan, Aparecido A. Camacho, Juliana A. Cerqueira, Evandro Zacché, Murillo D. Kirnew, Bruna A. Loureiro, Samara B. Silva, Henriette G. Moranza, Guilherme C. Ferraz

**Affiliations:** 1 Department of Animal Morphology and Physiology, Laboratory of Pharmacology and Equine Exercise Physiology (LAFEQ), School of Agricultural and Veterinarian Sciences (FCAV), São Paulo State University (UNESP), Jaboticabal, SP, Brazil; 2 Department of Veterinary Medicine and Surgery, School of Agricultural and Veterinarian Sciences College of Agriculture and Veterinary Sciences (FCAV), São Paulo State University Universidade Estadual Paulista (UNESP), Jaboticabal, SP, Brazil; 3 Centro de Ciências Agrárias, Department of Animal Science, Universidade Federal da Paraíba, Centro de Ciências Agrárias, Areia, Paraiba, Brazil; West Virginia University, UNITED STATES

## Abstract

The dogs’ responses to training exercise are seldom monitored using physiological variables, and cardiac autonomic regulation (CAR) is a relevant determinant of endurance-training adaptation. There are studies in the literature establishing that regular exercise could interfere with CAR in dogs, measured by heart rate and vagal-derived indexes of heart-rate-variability (HRV). However, few studies were found using a prescribed training program based on the lactate threshold (LT) to determine HRV by a 24-h Holter analysis. The purpose of this study was to test whether an endurance-training program (ETP) guided individually by LT raises time-domain measures of HRV in healthy Beagle dogs. Twenty dogs were assigned to two groups: control (C) and trained (T). The dogs from group T underwent an incremental exercise test (IET) to determine their LT. Both LT and velocity corresponding to the LT (VLT) was determined by visual inspection. T group performed an eight-week endurance-training program consisting of treadmill runs set to 70–80% of the VLT. Next, dogs from the group T have submitted to IET again. The maximal velocities (Vmax) at which achieved by the trained dogs in both IETs were determined. The group S did not undergo IETs or ETP. HRV was determined by the 24-hour-Holter at rest, before and on the 2°, 4°, 6° and 8° training weeks. To examine the HR impact on HRV, standard HRV variables were normalized to prevailing HR. VLT and Vmax rose in group T, indicating an improvement of dogs’ aerobic and anaerobic capacity. The normalized standard HRV indexes were relatively attenuated since these variables had a reduction in the degree of correlation concerning an average HR. The ETP resulted in decreased resting heart rate and increased time-domain indices, highlighting the log-transformed square root of the mean sum of the squared differences between R–R intervals (Ln rMSSD). The lactate-guided endurance-training program could lead to better parasympathetic cardiac modulation in Beagle dogs.

## Introduction

Efficient exercise training provides a stimulus to increase performance, and continuous monitoring of athletes may supply information that could be used to prescribe running intensity. Therefore, commonly used monitoring tools are exercise tests [1], blood blood-borne markers such as lactate [2–3], and heart rate (HR)-based measures for monitoring the status of the autonomic nervous system (ANS) [4]. Ideally, to be used frequently in sports, these methods should be quick to apply, inexpensive, and sensitive to diagnose performance changes. A recent study performed to check some adjustments in cardiovascular performance induced by exercise in healthy military dogs, reports that monitoring HR in dogs during exercise on a treadmill is a promising advance in veterinary cardiology [5]. Owing to scientific progress and greater consensus on methodology, evaluation of heart rate variability (HRV) has become non-invasive and more available for assessment in recent years.

Heart-rate-variability (HRV) is the fluctuation of time intervals between adjacent heartbeats that can be used to indirectly detect ANS condition in athletes humans and animals [6–8]. Such a tool has determined from time-domain indices, which is the time period between successive heartbeats obtained through statistical calculations based on R-R intervals [6]. This method may provide relevant data on the ANS of healthy [8–10] or infirm animals [11,12]. Also, HRV is used as a marker of performance of executive functions like attention and emotional processing by the prefrontal cortex in human patients [7–13], and some external factors may influence HRV. For example, HRV measurement may be influenced by circadian rhythm and the environment [6]. For this reason, 24-hour HRV recordings are considered the "gold standard" for clinical evaluation of HRV in veterinary and human medicine [6, 8]. For the past two decades, HRV has been used to determine fitness effects on ANS in healthy humans [14–17], or affected with cardiovascular disease [18]. These studies have shown that chronic exercise could increase both cardiac autonomic regulation (CAR) and parasympathetic activity, and decrease sympathetic tonus [14].

Although some studies in dogs have established that exercise training could lead to reduced resting heart rate (HR_rest_) while increasing vagal HRV, few have accurately reported the intensity of effort used as a guide for prescribing the fitness protocol. These studies used healthy dogs [19] or dogs affected by experimentally induced myocardial infarction through coronary artery occlusion [20,21]. Furthermore, the HRV determined in these previous studies was obtained by electrocardiography immediately before or during 30-s to 5-min after exercise. However, a recent review stated that HRV obtained in short or ultra-short HRV measurements (seconds or minutes) could bring markedly different physiological results compared to methodologies using Holter-24h [6]. Despite this fact, to the best of our knowledge, the literature on dogs lacks research focusing on HRV obtained using Holter-24-h and lactate threshold training.

Because trained athletes have higher HRV than sedentary individuals, it has been suggested that exercise training could increase heart rate variability in normal populations.The HRV of dogs submitted to chronic exercise was studied in conditioning programs prescribed based on estimated effort intensities, with a relative degree of subjectivity, between 70% and 80% [20,21] or 60 to 80% [19] of the maximum heart rate (HRmax). These authors reported divergent findings while the first study reported an increase in HRV, the second found no change. Such conflicting results may be possibly related to the methodology used to guide the training effort intensity or the HRV determination method that used Holter immediately before and during the exercise test [21] or electrocardiogram at rest [19]. Additionally, other factors may interfere with results such as different conditioning stages, difficulty in determining actual resting state, or lack of familiarity of the dogs with the laboratory facilities and the procedure itself as well [22].

Some studies in humans and have demonstrated that lactate-threshold intensity, which distinguishes domains of moderate to high-intensity exercise, may lead to distinct cardiovascular and metabolic responses postexercise recovery period. Also, it has been established that exercise training may induce a diversity of physiological adjustments in dogs, according to the breed, duration, intensity of training program and fitness level [2, 3, 5]. Although most dogs have a quite active life, still there is a real need for structured exercise programs. First, most small breeds are predisposed to cardiovascular disorders such as myxomatous mitral valve disease, which has a higher prevalence in smaller (<20 kg) dogs [23]. Thus, from the substantial health-enhancing potential of physical activity [19], active dogs, including Beagle dogs, even those sedentary individuals, can incorporate forced regular aerobic exercise on a treadmill at least a moderate intensity into their daily life. Furthermore, it is well known that Beagles are often used as experimental models in Veterinary Medicine, as well as in the studies in Medicine. Also, the research findings on the ‘*threshold-based model*’ approach to prescribing exercise intensity have been extrapolated to other breeds.

In this sense, studies in dogs, focusing on the action of an aerobic-training program on HRV, with deterministic prescription of intensity, expressed as running pace in meters per second, could be considered a gap in the dogs’ literature. In sports medicine, it is common to use incremental exercise tests (IET) to obtain the lactate curve, which is a classic parameter commonly used to diagnose aerobic capacity and guide training intensities [2,3]. Also, there are few published research findings on using LT as a guide for the exercise velocity for training in dogs. The present study tested the hypothesis that improvement in aerobic exercise capacity resulting from endurance-training program (ETP) increases the HRV time-domain measures. Based on these considerations, this study aimed to evaluate the HR and HRV in Beagle dogs that underwent an eight-week ETP. HRV was determined using “Holter 24-h” and the computed time-domain measures included standard deviation of NN intervals (SDNN), root mean square of successive RR interval differences (RMSSD), percentage of successive RR intervals that differ by more than 50 ms (pNN50), and the natural log of the (Ln RMSSD).

## Material and methods

The study followed the Ethical Principles in Animal Experimentation adopted by the Brazilian College of Animal Experimentation and approved by the Ethics Committee on Animal Use (CEUA-FCAV/Univ Estadual Paulista) under protocol 008272/17.

### Research site

The experiment was conducted at the Equine Exercise Physiology and Pharmacology Laboratory (LAFEQ) and the Laboratory of Cardiology of the Veterinary Hospital of the Universidade Estadual Paulista (UNESP), in Jaboticabal, Brazil.

### Dogs

Twenty clinically healthy Beagles, between 12 and 24 months of age, were used in this study. The dogs belonged to the kennel of the Laboratory of Nutrition and Nutritional Diseases of dogs and cats of the School of Agrarian and Veterinary Sciences (FCAV), UNESP, in Jaboticabal, SP, Brazil. All dogs were semi-sedentary and had no previous forced training exercise on a treadmill. The dogs were considered healthy based on their medical history, physical examination, bloodwork, and 2-dimensional, M-mode and Doppler echocardiography (Acuson X300 Ultrasound System, Premium Edition, Siemens, Munich, Germany). Exclusion criteria included any alteration of bloodwork results and possible clinical abnormalities that could affect exercise performance, particularly cardiovascular, neuromuscular and orthopedic conditions.

The dogs lived in pairs in 1.5 x 4.0 m kennels with a solarium and daily access to an outside 1000-m^2^ playground for exercising and socializing for 4 hours a day. These housing, management, and care were necessary to maintain their well-being. The dogs were fed commercial rations to meet their energy requirements equivalent to active kennel dogs, according to NRC [24]. Water was available *ad libitum*. During the experiment, the dogs were not fed 12 h before exercise while on training days, the feed was supplied 20 minutes after the exercise. The dogs were weighed every fifteen days over the entire experimental period, and when necessary, feed amount was adjusted to maintain animal body weight.

### Treadmill adaptation

The followed treadmill adaptation protocol has been previously used and reported by our laboratory [3]. For ten consecutive days, the dogs were allowed to adapt to the laboratory where the IETs and the ETP were going to be performed. The dogs were encouraged to climb on the powered-off treadmill (Galloper® 5500, Sahinco), which was then turned on and set at an initial speed of 1.5 m/s (5.4 km/h), forcing the dog to move, initiating safely and effectively the adaptation to the different speeds. For positive reinforcement, the dogs were rewarded with biscuits after the exercise sessions.

### Experimental groups

The dogs were distributed into two groups. The trained dogs were submitted to two maximal incremental exercise tests (IET-1 and IET-2) separated by an eight-weeks ETP (n = 12, 6 males and 6 females—trained group, T). Dogs in the control group did not participate in any forced exercise (n = 8, 4 males and 4 females—control group, C), being provided access to a collective outdoor playground. This C group was used to verify possible interference of environmental factors such as behavioral and emotional factors.

### Incremental exercise test

The IETs were performed to assess the functional capacity of the dogs so that individual ETP could be prescribed for the T group. The test protocol was adapted from previous studies conducted by our research group [2,3]. Once adapted to the treadmill, dogs were submitted to the following protocol, warm-up starting at 1.5 m/s (5.4 km/h) initial speed and 0% slope, subsequent speed increments of 0.5 m/s (1.8 km/h) at 7.5% slope. Speed was increased every 5 min until dogs showed signs of fatigue and inability to keep up the treadmill velocity belt despite positive reinforcement by a staff member positioned in front of the treadmill encouraging the dogs when needed (see **S1 Video** for details). It must be highlighted that no dog was submitted to negative reinforcement, such as shock or something similar. IETs were always performed in the morning between 8 a.m. and 12 p.m., in a room with a temperature ranging from 19 to 22 ºC. All dogs underwent 3-h fasting with free access to water. The IET was performed to obtain the lactate velocity curve and the LT, which was related to the speed achieved on the treadmill, thus establishing the velocity correspondent to the lactate threshold (VLT). All dogs of group T were submitted to this step to set up the ETP velocity. The IET was repeated after eight weeks to compare the VLTs obtained in IET-1 and IET-2. Also, the anaerobic power was determined at the maximal velocity (Vmax) of the IETs. For VLT and Vmax baseline values and changes in the untrained group (control dogs) after the ETP period, see our recently published study [2].

### Determining the lactate threshold (LT)

**Fig 1:**
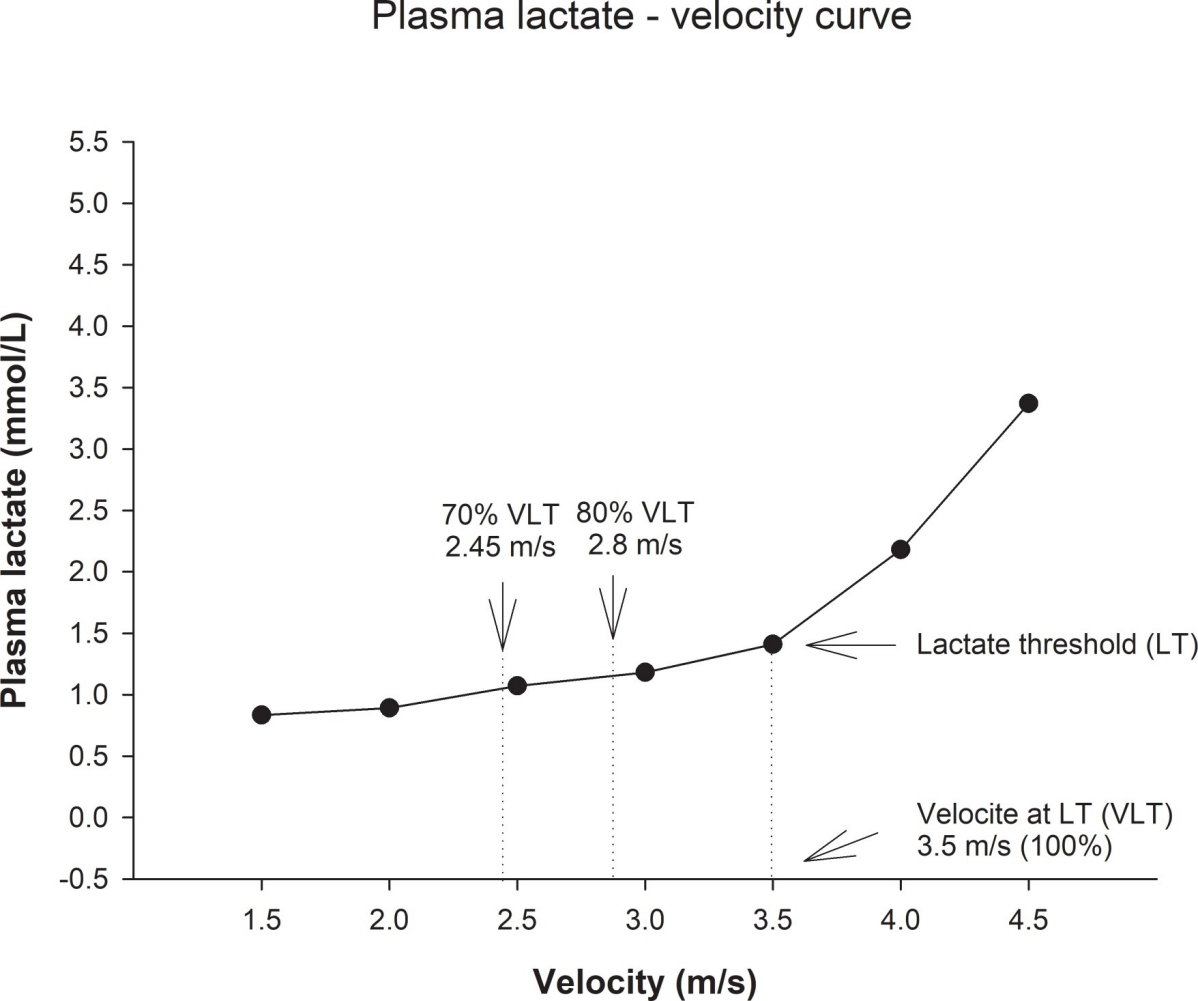
Graphical representation of the typical lactate-velocity curve (LVC) of a representative single Beagle dog submitted to the first incremental exercise test (IET-1). Plasma lactate concentration (mmol/L) vs. velocity (m/s). The increase in plasma lactate concentration corresponds to the lactate threshold (LT). The velocity at the lactate threshold (VLT) was used to prescribe the endurance-training program (ETP) intensity.

For detection of LT, a series of venous blood samples were drawn during IETs. Three evaluators determined the LT through a visual analysis [2] of the lactate-velocity curve (LVC), obtained in the IET-1, to identify the speed in which the plasma lactate concentration presents an abrupt and exponential increase [3]. The deflection point represents the start of an imbalance between lactate production and removal/metabolism (Fig 1). We applied the exponential function y = ae^bx^ ⁠(where y = lactate concentration, mmol/L and x = velocity, m/s) to verify the mathematical standard of the LVC [2]. The VLT obtained in IET-1 was used to prescribe the individual training intensity.

### Blood sample

At each IETs speed step, a blood sample (2-mL each) was collected via venous catheterization of the jugular vein using a 16G InsyteTM catheter (Becton Dickinson and Company Belliver Industrial Estate, United Kingdom). Topical skin anaesthesia based on 2.5% lidocaine and 2.5% prilocaine (EMLA®) was applied to the trichotomized area. The catheter was held in place with a drop of Super Bonder® to ensure its fixation. Blood (4 mL) was drawn after 1.5 min of passive rest following each incremental stage of the IETs, as performed in a previous study [3]. Venous blood samples were packed in tubes containing EDTA (12 mg) and sodium fluoride (6 mg) (BD Vacutainer® Fluoride/EDTA, (Becton Dickinson and Company Belliver Industrial Estate, UK) to determine the lactate concentration. The plasma was separated by centrifuging the samples at 1470g at 4°C for 10min. After this, the electro-enzymatic methodology (YSI 2300, Yellow Springs Instrument, USA), previously validated for use in dogs [25], was used.

### Endurance training program

As mentioned above, a first incremental exercise testing (IET-1) on the treadmill was conducted after the adaptation period to establish the LT from the LVC for each dog. All dogs in the T group completed eight weeks of aerobic exercise. So, between the IET-1 and IET-2, the T group underwent an eight-week ETP and supervised exercise sessions were offered three times a week (Mondays, Wednesdays, and Fridays) between 8 a.m. and 12 p.m. The intensity of the conditioning was prescribed at 70% and 80% of the VLT at 7.5% slope, 70% in the first 4 weeks and 80% from week 4 onward. Each exercise session consisted of 5 min of warm-up at 50% of the VLT, 20 minutes of exercise at the prescribed speed (70 or 80% of VLT) and 5 minutes of cooling down (50% of the VLT, again). After this period, the dogs underwent another incremental exercise test (IET-2). It is noteworthy that the dogs of group C performed 30 min of recreational activities in the kennel facilities, being stimulated with positive reinforcements in the stall (snacks) 3 times a week. Only dogs that attended 100% of the exercise training sessions (T group) were included in the final study sample. C group did not participle in the exercise training program but maintained their current lifestyle. All dogs were evaluated to obtain the HR and HRV.

### Holter electrocardiography and timepoints

The digital Cardioflash® equipment (Cardios Sistemas, São Paulo, Brazil) was used to monitor cardiac electrical activity for 24-h and to determine HRV, and the signals were captured by 2223BRQ® adhesive electrodes (3M São Paulo, Brazil). The Holter 24-h was obtained with 4—way and 3—channel electrode system, using a digital recorder. The electrode placement area was previously trichotomized, and the skin was cleaned with alcohol. The connections were arranged to approximate leads I, II and III [3,12]. A bandage was applied to the thorax of dogs that wore a vest for storing the digital recording device. Electrocardiographic tracings were processed by specific software (CardioMananger S540, Cardios Sistemas, Brazil). Only recordings with > 23 h and < 3% artifacts were included, initially processed and automatically analyzed by commercially available software, then, manually reviewed for arrhythmias and artifacts, and verified for proper classification of each QRS complex by a Veterinary Cardiologist (A.Z.R). During the 24h-Holter examination, the dogs were always kept with the same dog in the same stall during each HRV assessment to avoid changing environmental variables that could interfere with the results of the 24h examination. Importantly, all animals were acclimatized to the environment and the daily activities of the kennel.

HRV variables were determined in the time domain, quantifying standard deviation of NN intervals (SDNN), standard deviation of the average NN intervals for each 5 min segment of a 24-h HRV recording (SDANN), root mean square of successive RR interval differences (RMSSD), and percentage of successive RR intervals that differ by more than 50 ms (pNN> 50). Also, natural logarithm (Ln) was used for the RMSSD variable (Ln RMSSD) to obtain a normal distribution. To lessen the impact of HR on HRV parameters, Pearson’s correlation was applied to the HRV calculations and average HR. This normalization (i.e., removing both mathematical and physiological HRV dependence on HR) was performed from the previous studies [26,27]. When the HRV and HR parameters were negatively correlated, they would be divided by suitable powers of their corresponding average R-R intervals. However, for a positive correlation, the correction relied on multiplication by adequate powers of average R-R intervals.

Readings were obtained in the baseline week (W0) and every 2 weeks (W2, W4, W6, and W8). All tests were performed 24-h after the conditioning session held in the week for group T, to mitigate any examination changes resulting from the exercise performed the day before [28]. The tests were performed in the dogs of both groups at the same place. Also, heart rate (HR_mean_), minimum heart rate (HR_min_), and maximum heart rate (HR_max_) were determined.

### Statistical analysis

For statistical analysis, all variables were checked for normal distribution by the Shapiro-Wilk test. Mann-Whitney test or Student t-test for unpaired samples was performed for the physiological characteristics of the groups. Fisher's exact test was used to evaluate the distribution of groups (sex category, males vs females) (see S1 Fig for details). The relationship between baseline HR and HRV with and without HR correction was evaluated employing linear regression and Pearson correlations coefficient *r*. The data were analyzed using a two-factor mixed-design ANOVA [group (Two levels: control, trained) x time (five levels: W0, W2, W4, W6, W8)] with repeated measures on one factor [time] measures. Post-hoc comparisons of the data were then carried out using a Tukey-Kramer multiple comparison test. The VLT and Vmax of IET-1 and IET-2 for group T were compared by the Wilcoxon Signed Rank Test. Statistical analyses were performed using SigmaPlot v.12.0 software at 5% significance level.

## Results

Table 1 summarizes the variables that describe the profile, indicating the cardiac health of the dogs in the experimental groups. The data from the two sexes were combined since no significant differences were no significant differences between their initial physiological characteristics at the ETP onset. In the present study, one dog (female) in group T had difficulty adapting to the high speeds during the ETP and was removed before completing the 8 weeks of training. Therefore, only the data of 5 female dogs of group T were available at the end of the study, so that a total of 19 out of 20 participants were included in the final analysis. The dogs’ weights of the T and C groups differed neither before nor after the experimental period. It is essential to add that none of the dogs showed changes in rhythm (ventricular or supraventricular arrhythmias) during the study.

**Table 1:** Baseline physical characteristics and cardiac measures of the dogs.

Variable	C	T	p
**Age (months)**	12.5 (12–20)	13 (12–24)	0.6^‡^
**Weight, Kg**	12.44±1.1	11.38±1.4	0.1*
**LA:Ao**	1.27±0.1	1.24±0.1	0.603
**LVIDd:Ao**	1.83±0.1	1.82±0.2	0.93*
**LVIDs:Ao**	1.12±0.1	1.2±0.2	0.27*
**FS (%)**	37.54±6.2	34.20±4.9	0.207*
**E:A**	1.68±0.2	1.74±0.3	0.631*
**HR (bpm)**	89 (82–104)	99 (82–117)	0.136^‡^

* Student t-test.

‡ Mann–Whitney test. ‡ ‡ Fisher exact test; LA:Ao: Left atrium-to-aorta ratio; LVIDd:Ao: Left ventricular internal dimension at end-diastole-to-aorta ratio; LVIDs:Ao: Left ventricular internal dimension at end-systole-to-aorta ratio; FS: Fractional shortening, E:A: ratio of early-to-late left ventricular filling velocities; HR: heart rate; T: training group; C: control group.

**Fig 2:**
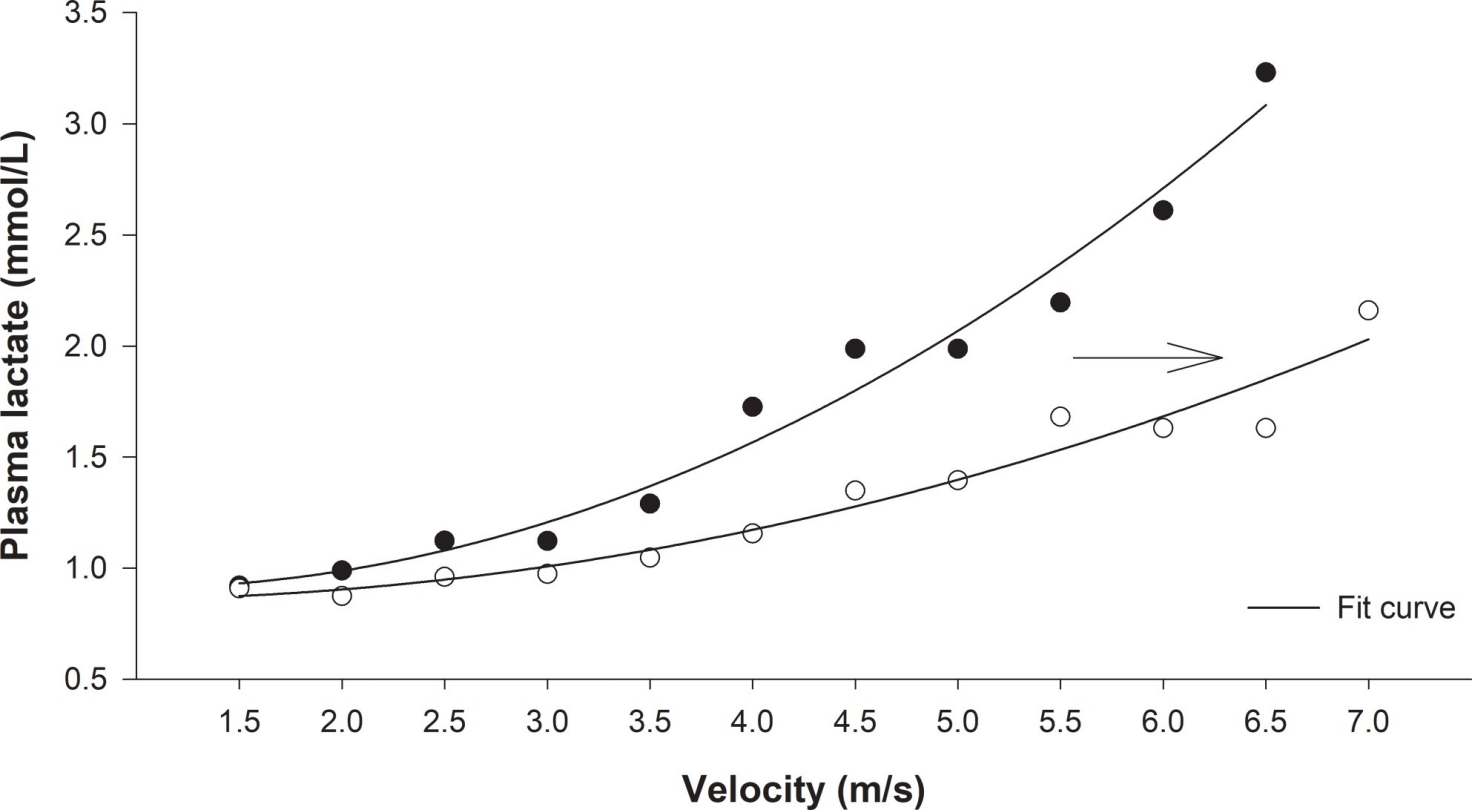
Lactate velocity curves (LVC) of Beagle dogs subjected to incremental exercise tests (IETs). LVC IET-1 and IET-2, after eight-week endurance-training program (Trained Group). Note the exponential tendency lines. IET-1: Y = 0.1341^.0706x^ ⁠, P<. .0001, R⁠ = 0.98; IET-2: Y = 0.0477^.0303x^, P < .0001, R⁠ = 0.97. Black and white dots are means and correspond to the values obtained at IET-1 (before training) and IET-2 (after training), respectively.

**Fig 3:**
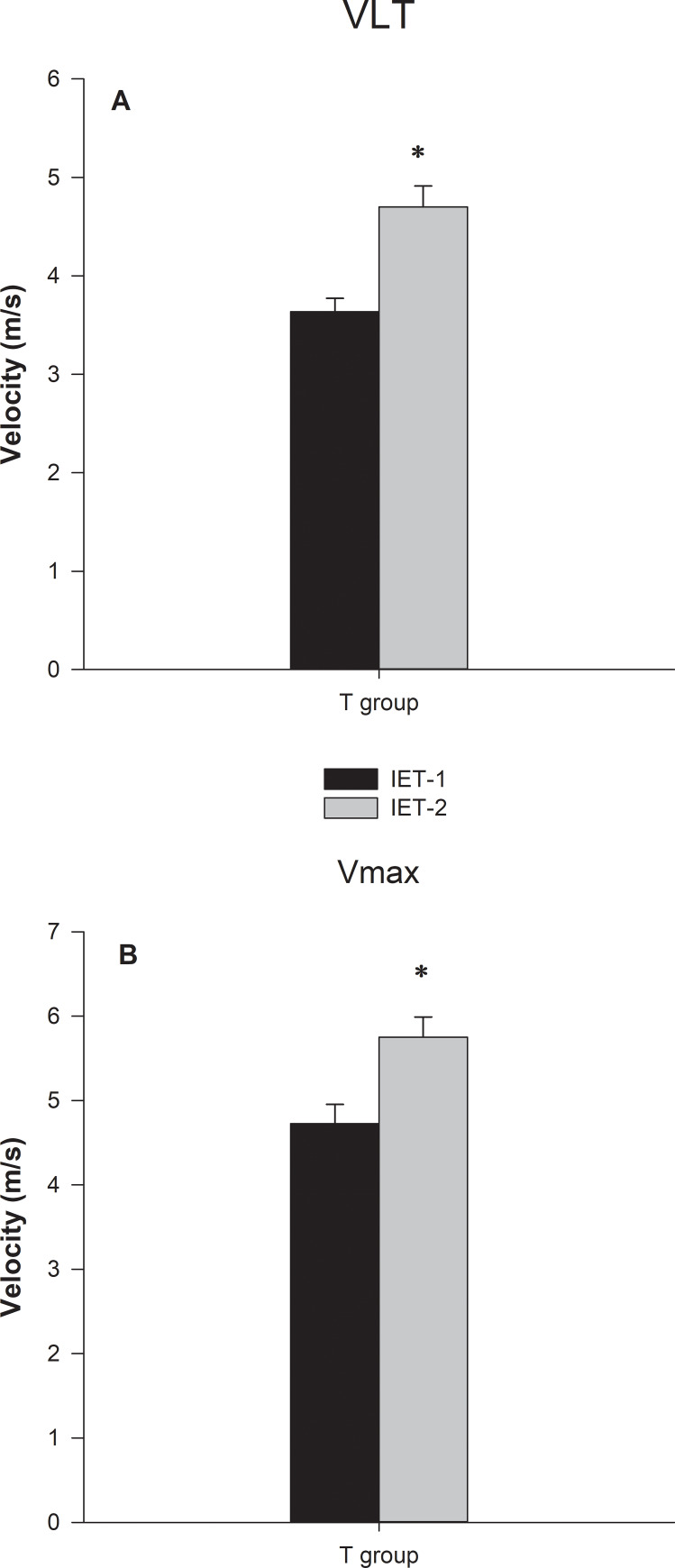
Graphical representation of the means ± standard error of (A) velocities at the lactate threshold (VLT) and (B) maximal velocity reached by Beagle dogs in the two incremental exercise tests (IETs). Dogs from the trained group. * indicates an increase in VLT and Vmax during the IET-2 in comparison with IET-1 (P < .05). Bar whiskers represent the standard error of the mean. For baseline and changes in VLT and Vmax in the untrained group after the ETP period, please see our recently published study [2].

**Fig 4:**
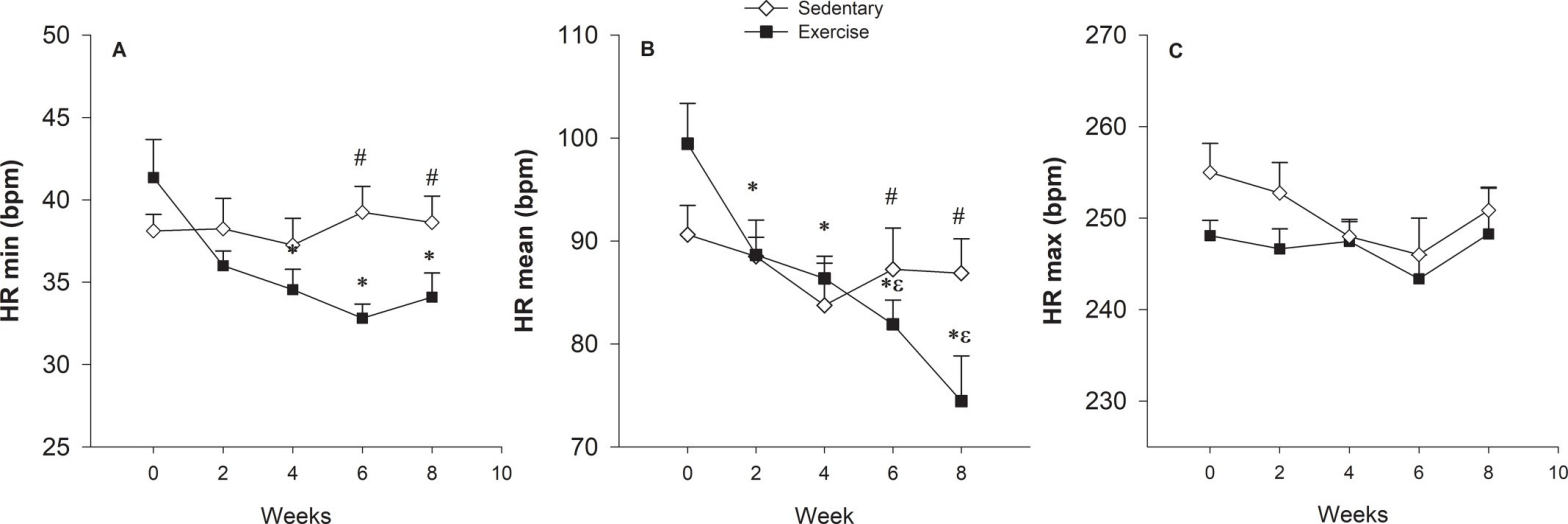
Changes in minimum heart rate (HR_min_) (A), mean heart rate (HR_mean_) (B), and maximum heart rate (HR_max_) (C). Bars are standard errors. # Indicates difference between groups; * Indicates difference in relation to week 0; ^ε^difference in relation to week 2 and 4. (p < 0.05).

**Fig 5:**
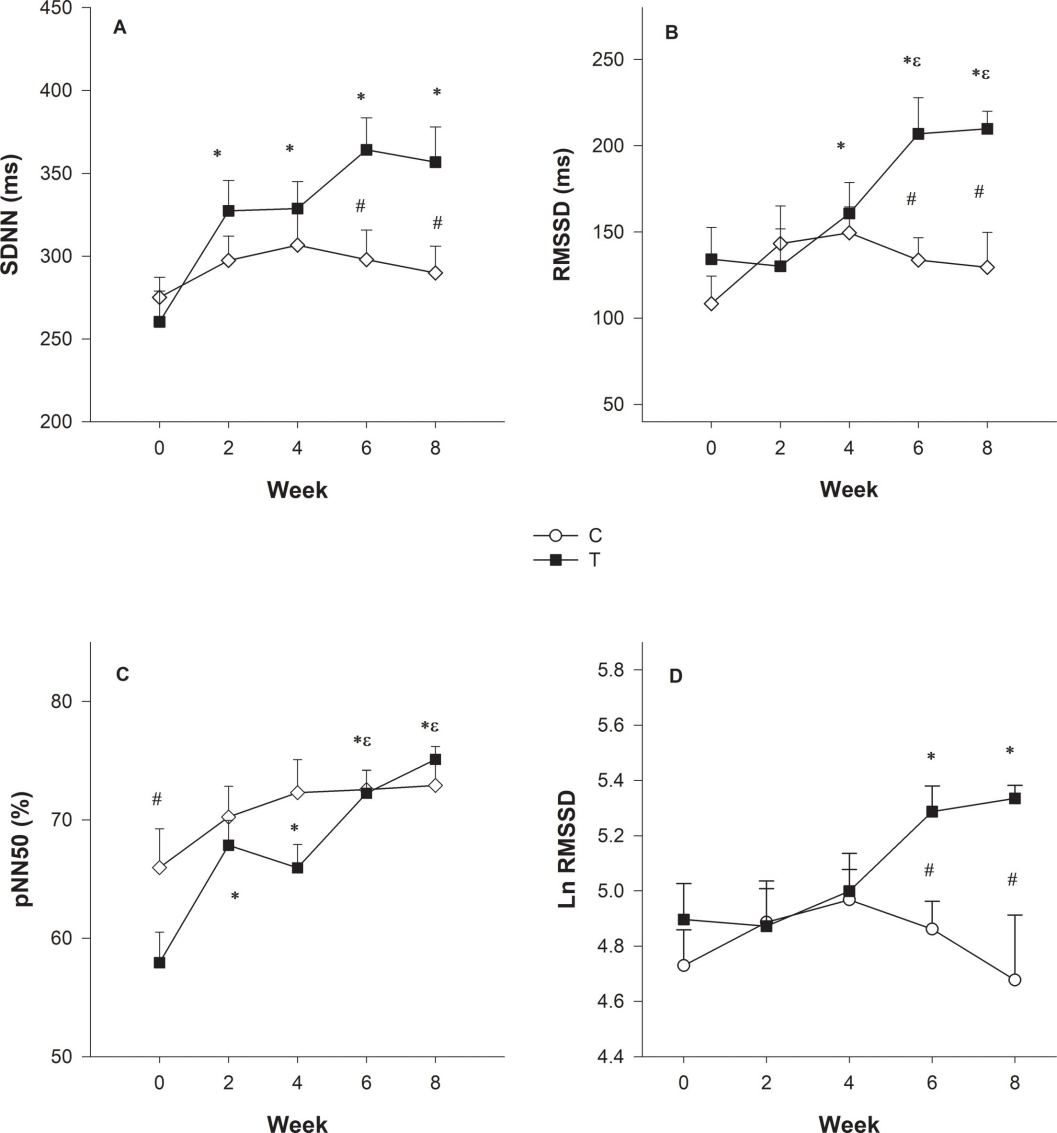
Changes in time-domain measures on heart rate variability (HRV) in dogs undergoing 8-week physical training. SDNN: standard deviation of all normal RR intervals (A); RMSSD: the square root of the mean of the squared differences between adjacent normal RR intervals (B); pNN50: percentage of differences between adjacent normal RR intervals that are >50 ms (C); LnRMSSD: natural logarithm of the square root of the mean sum of the squared differences between R–R intervals (D). Bars are standard errors. # Indicates difference between groups; * Indicates difference in relation to week 0; ^**ε**^ difference in relation to week 2 and 4 (p < 0.05).

We used velocity related to plasma lactate concentration to prescribe and evaluate the ETP efficacy. The heart rate values at the initial training running velocity (70% VLT) and after four weeks (80% VLT) corresponded to the 154±10 and 176±11 bpm, respectively. The trend curves and their equations for the trained dogs revealed that their LVC followed an exponential growth mathematical model (P < .0001) (Fig 2). The comparison between IET-1 and IET-2 of trained dogs indicated that both aerobic and anaerobic capacities increased, keeping in mind that VLT and Vmax were higher for IET-2 compared to IET-1 (P = 0.002) and (P = 0.008), respectively (Fig 3). Before and after the ETP, mean VLT (± SD) values were 3.63 ± 0.13 and 4.70 ± 0.21 m/s, respectively. Vmax values were 4.72 ± 0.22 and 5.75 ± 0.23 m/s for T group. In the untrained group, the VLT and Vmax did not differ between the IETs (please see [2]; in https://doi.org/10.1016/j.rvsc.2018.10.004). The VLT and Vmax variables were no significantly different between sexes over time due to the ETP. In females, the mean VLT (± SD) were 3.80 ± 0.7 and 4.60 ± 0.6 m/s; and Vmax values were 5.0 ± 1.0 and 5.2 ± 0.5 m/s, before and after, respectively. In males, the mean VLT (± SD) were 3.60 ± 0.2 and 4.50 ± 0.4 m/s; and Vmax values were 4.7 ± 0.4 and 5.9 ± 0.6 m/s, before and after, respectively. The HRs related to the VLT were 221±14 and 231±16 bpm, being corresponding to the 91.7% and 94.6% of HR_max_. The HRs corresponding to the Vmax were 241 ± 10 and 244± 14 bpm for trained dogs. In the entire study (Holter 24-h), the HRmean and HRmin values were affected by training, while a significant group vs. time interaction was detected for HRmean (p = 0.001) and HRmin (p = 0.004). The HRmax values were not affected by training (Fig 4). For the resting HRV values of SDNN (p = 0.008), SDANN (p = 0.008), RMSSD (0.013) and pNN50 (p = 0.02), there was a significant interaction group vs time (Fig 4). Time (weeks) affected mainly Ln RMSSD (p = 0.016) so that there was no difference between groups (p = 0.15). Post-hoc analyses indicate that only in T group, Ln RMSSD was significantly higher in week 6 (p = 0.005) and 8 (p = 0.002) compared to both baseline and week 2 (p = 0.019) and (p = 0.007), respectively (Fig 5).

**Fig 6:**
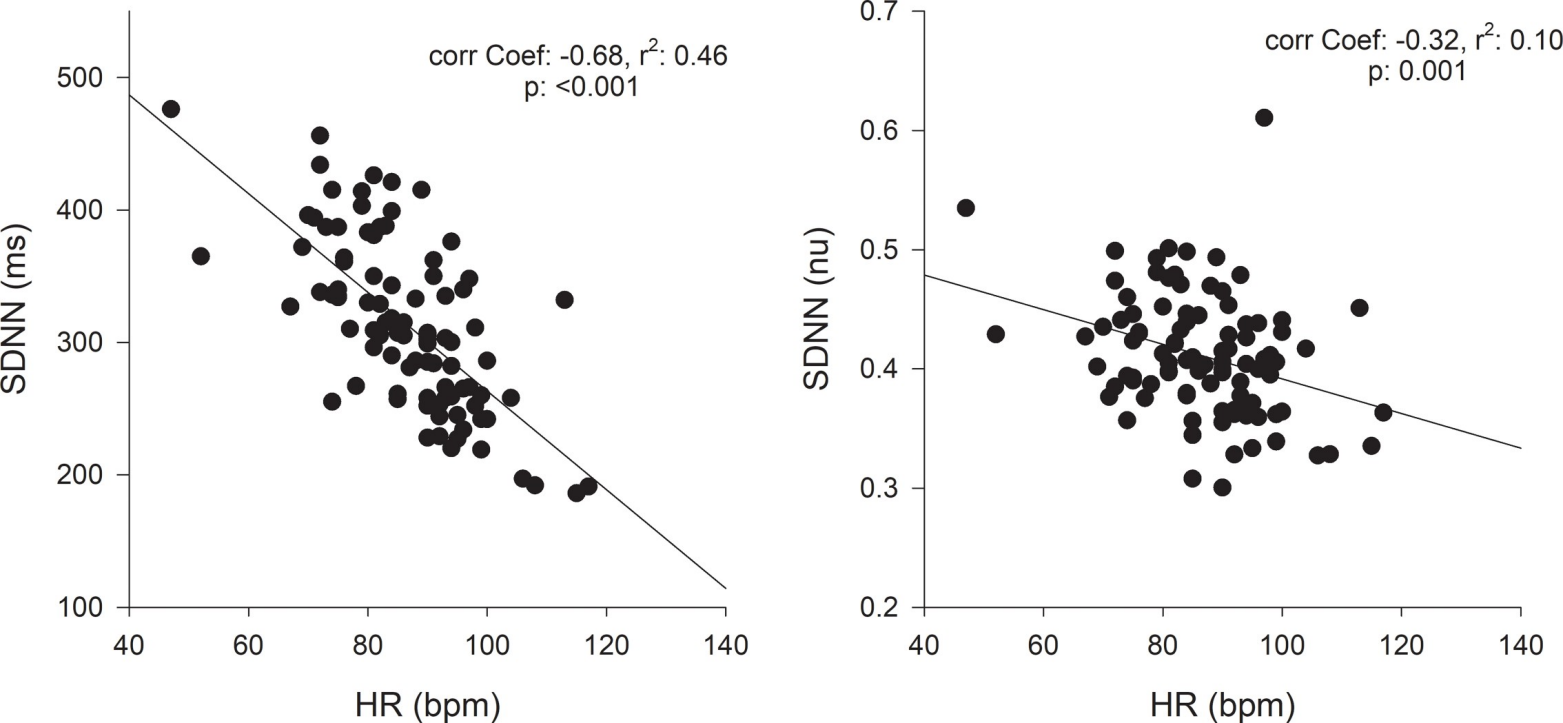
The relationship between mean HR (HR_mean_) and heart rate variability (HRV). SDNN: standard deviation of all normal RR intervals. One data point is displayed for each dog Holter (n = 95). The data without and with HR correction (SDNN/mean RR) are displayed in A, respectively. Note that HR only accounted for than 46% (r^2^ = 0.46) of the variability before correction for HR. nu, normalized units following HR correction.

**Fig 7:**
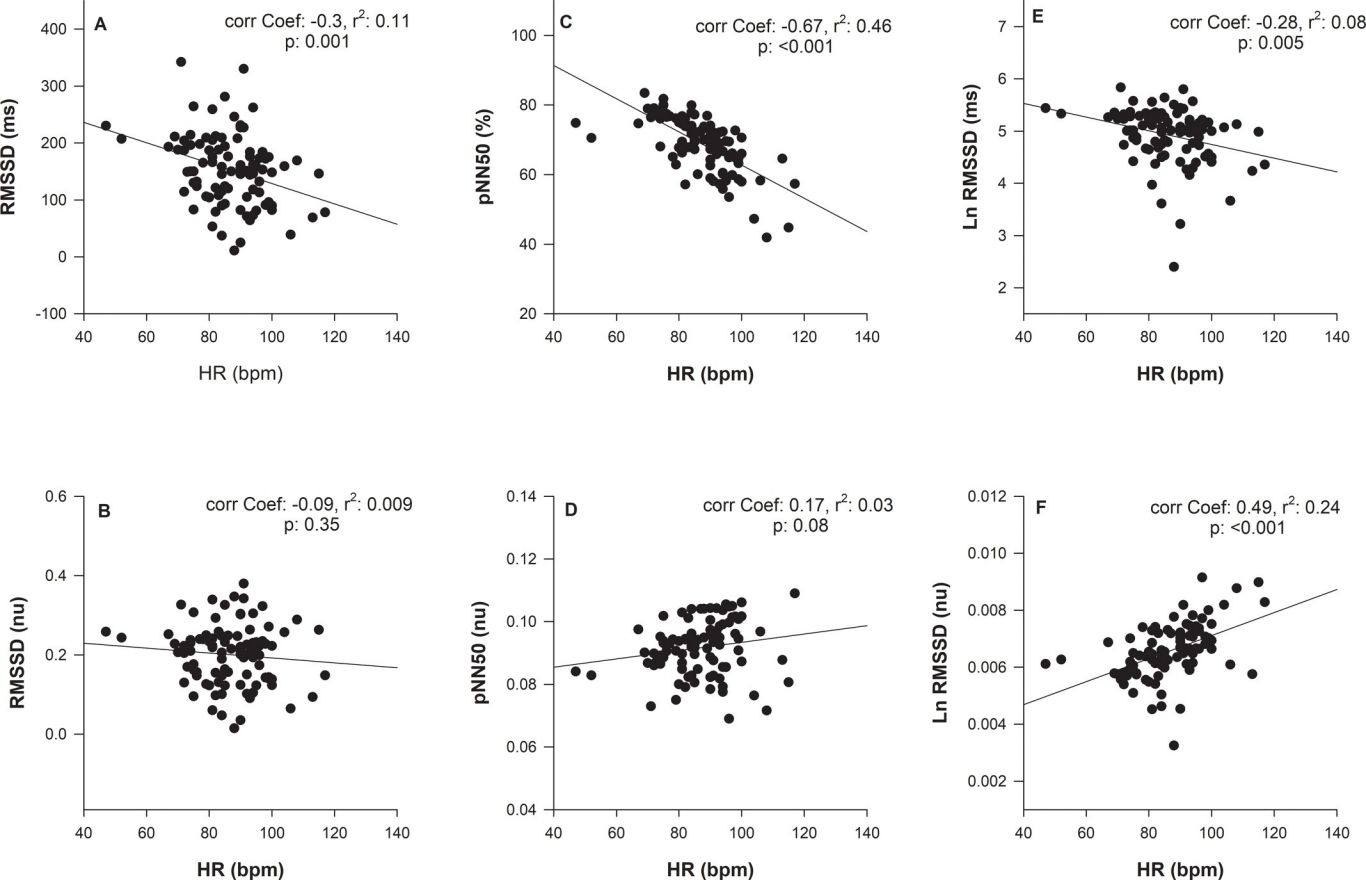
The relationship between mean HR (HR_mean_) and heart rate variability (HRV). RMSSD: the square root of the mean of the squared differences between adjacent normal RR intervals (A); pNN50: percentage of differences between adjacent normal RR intervals that are >50 ms (C); LnRMSSD: natural logarithm of the square root of the mean sum of the squared differences between R–R intervals (E).

The Figs 6 and 7 show that SDNN, RMSDD, pNN50 and Ln RMSSD were negatively correlated with HR and the correlation coefficients ranged between −0.28 to −0.68 (n = 94; p < 0.01 for all). Correction for the prevailing HR suppressed the HR impact on SDNN (Pearson’s correlation coefficient = −0.32, p = 0.001; Fig 6B), RMSSD (Pearson’s correlation coefficient = −0.09, p = 0.35; Fig 7B), pNN50 (Pearson’s correlation coefficient = 0.17, p = 0.08; Fig 7D), and Ln RMSSD (Pearson’s correlation coefficient = 0.49, p< 0.001; Fig 7F).

**Fig 8:**
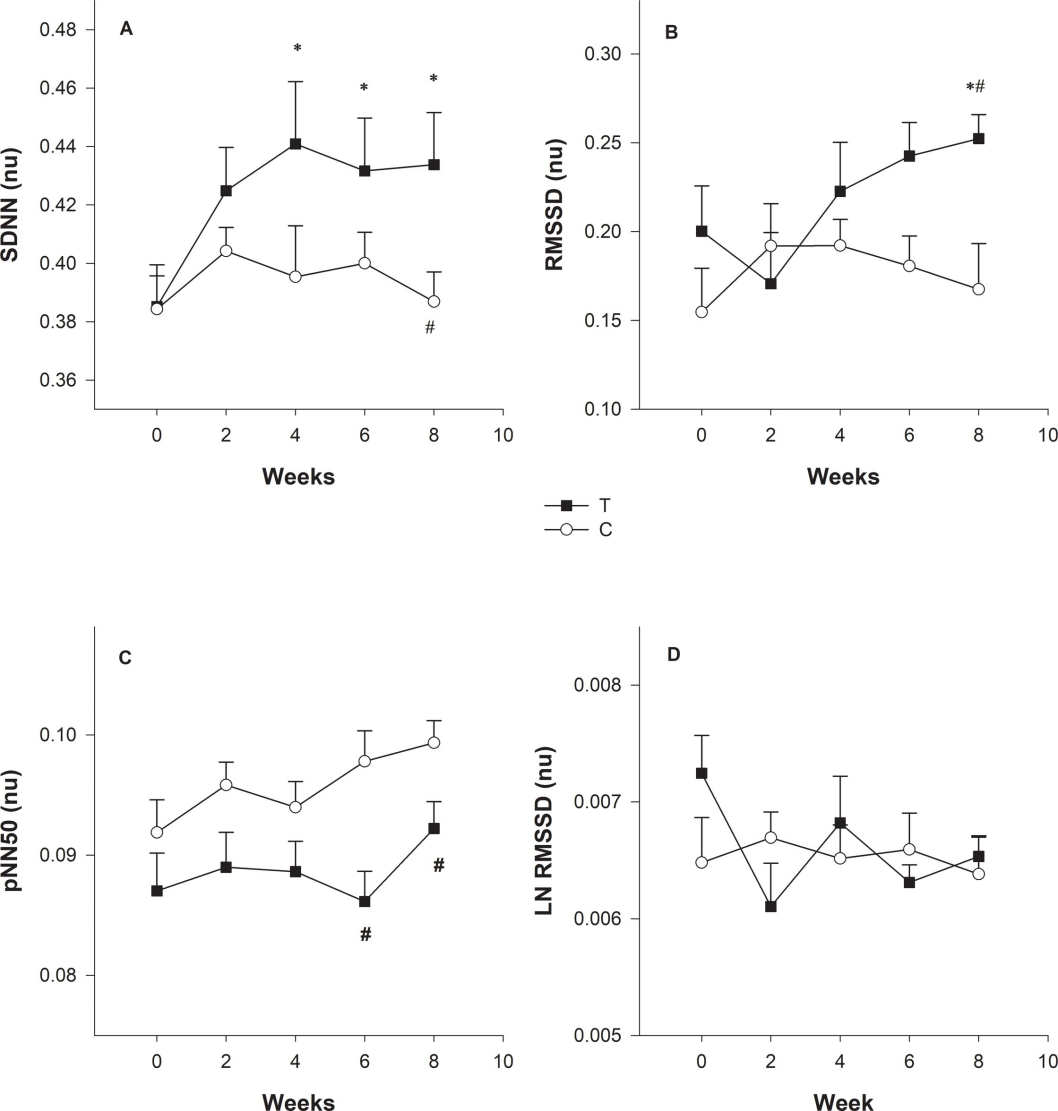
Changes in time-domain measures on heart rate (HR_mean_) and heart rate variability (HRV) in dogs undergoing 8-week physical training. SDNN: standard deviation of all normal RR intervals (A); RMSSD: the square root of the mean of the squared differences between adjacent normal RR intervals (B); pNN50: percentage of differences between adjacent normal RR intervals that are >50 ms (C); LnRMSSD: natural logarithm of the square root of the mean sum of the squared differences between R–R intervals (D). The data without and with HR correction (RMSSD/meanRR, pNN50/meanRR and Ln RMSSD/meanRR). nu, normalized units following HR correction. Bars are standard errors. # Indicates difference between groups; * Indicates difference in relation to week 0; (p < 0.05).

Fig 8 shows the results without and with HR correction. One data point was displayed for each dog Holter (n = 95). The normalization (nu, normalized units following HR correction) changed in the HRV indexes RMSSD/meanRR (Fig 8B), pNN50/meanRR (Fig 8C), and Ln RMSSD/meanRR (Fig 8D). For the T group, SDNN(nu) increased over eight weeks (p = 0.026), and in the fourth, sixth, and eighth week, the p values were 0.002, 0.012, and 0.008, respectively. In the eighth week, group T had higher SDNN (nu) value when compared with untrained dogs (p = 0.048). In the eighth week, group T had higher RMSSD (nu) values than untrained dogs (p = 0.045) and was higher (p = 0.025) in the intra-group comparison between the baseline (week 0) and eighth week. For pNN50(nu), there was a difference between groups over eight weeks (p = 0.007). However, after the correction pNN50 (nu), values have changed markedly, and group T had lower values compared to C group in the sixth and eighth week, and the p values were 0.003 and 0.05, respectively. There no difference between the groups (p = 0.70) for Ln RMSSD(nu).

## Discussion

One of the crucial issues in designing a training program for the dogs is prescribing an applicate exercise intensity. If routine training at lower intensities could raise aerobic capacity and fitness, it would be of considerable interest and importance in the prescription of regular exercise for health promotion in sedentary dogs. The main findings of the present investigation were that dogs subjected to the lactate-guided ETP displayed in increased HRV in the time domain and reduced baseline HR_mean_. Significant increases in SDNN, SDANN, RMSSD, and Ln-RMSSD were observed in dogs of group T. Interestingly, a new result of the current study was the increase in resting RMSSD, Ln-RMSSD, and pNN50 of dogs submitted to the ETP. No previous study has reported changes in these HRV measures in 24-h resting trained dogs. Also, the dogs used herein were a model to test the physiological changes to the exercise using 24-Holter electrocardiography that can be extrapolated to clinical populations.

The exercise prescription aimed to maximize safety, performance, and compliance with the ETP. Since the intensity of our ETP ranged from 70%-80% of VLT, it is anticipated that security was assured. This methodology was useful to change the HRV-related indexes positively in dogs, and previous studies have shown that conditioning programs using 70 to 80% of the VLT improved aerobic capacity in horses [29] and dogs [2]. The VLT of group T shifted to the right after the conditioning period, together with lower lactate concentrations for the same exercise intensity, suggesting less need for anaerobic glycolytic pathway and higher aerobic fitness. This result may be explained by the used training protocol, which probably increased the number of mitochondria and oxidative enzymes in muscle fibers, thus maximizing the oxidative phosphorylation pathway [29]. Additionally, VLT could be incremented by the familiarity or accommodation ability of the dogs to the treadmill that would represent an improvement of mechanical efficiency during the IET-2, since the velocity at lactate threshold is also dictated by the economy of movement [30]. Therefore, the methodology using a VLT-guided program for improving dog fitness could be a new tool to increase the species' aerobic conditioning. Moreover, the Vmax increase in group T in the IET-2 indicates anaerobic metabolic adaptation in response to conditioning. Importantly, it has been demonstrated by our published study [2].

Another aspect to be addressed was the reducted HR_mean_ indicated by the Holter 24h results. This result is the expected response after aerobic conditioning programs, and bradycardia during rest is used as a marker of cardiovascular improvement [21, 31,32]. However, in healthy dogs, the results observed in the literature were contradictory. Sled dogs performing light intensity exercise, between 30 to 40% of $\dot{V}O_{2}\max$ and 20 km daily average distance for 20 weeks have shown a reduction in HR [33]. Subsequently, this same study group investigated sled dogs submitted to a similar conditioning protocol and reported no change in HR [34]. These contradictory findings may be explained to some extent by the intensity of exercise employed while the continuous physical activity performed in a range lower than 50% of $\dot{V}O_{2}\max$ may not be enough to induce beneficial cardiovascular changes [15,17]. Another recent study found no difference in HR immediately before exercise testing after 6 weeks of conditioning. It is essential to bring to this discussion that HR in dogs obtained before the IET may suffer environmental interference, including excitement due to anticipation of exercise. Differences in the conditioning protocol and the time at which the HR_mean_ was obtained, as well as dog breeds used in the study and dog familiarization with both the research team and the laboratory environment, may interfere with the results obtained [19]. Also, during the night, the HRmin decreased in the T group. This finding could be explained due to parasympathetic activity predominance, which occurs across nighttime sleep, from 2 am to 6 am, a resting period of dogs. Generally, HRmin declines throughout sleep and appears when the parasympathetic is reaching its maximal activity relative to the autonomic nervous system. [35, 36]. These results could be used to explain the vagal tone improvement elicited by ETP, which was consistent with the progressive rise in cardiac parasympathetic activity.

In the literature, it is well known that environmental factors can interfere with the emotional and modify HRV in dogs [37]. It should be emphasized that the control group C, which was not trained, was used to verify whether adaptation to external interferences, during the experimental period, could potentially cause behavioural/emotional changes that resulted in reduced HR or increased HRV per se. To some extent, dogs had to get used and learn during the setup of the Holter device. However, this period of relative learning and adaptation to the environment was probably not enough to explain the results of HRV measures, whose indices improved significantly from the sixth week of conditioning in dogs of group T compared to group S. This result occurred concomitantly with the increasing effort intensity of the training protocol.

HRV is a noninvasive, practical and reproducible measure of autonomic nervous system function. Studies on humans [14] and canines [21] show that fitness programs prescribed at about 70% of HR_max_ increased the HRV after performing a fitness program on a treadmill for 10 to 12 weeks. Besides, a relatively shorter 8-week conditioning program, but with a gradually increasing intensity, increased the nocturnal HRV [17]. Similarly, in the present study, the HVR in trained dogs increased with the simultaneous increment of the intensity from 70 to 80% of VLT in the last four weeks. This finding corroborates results from other studies, which stated the importance of increasing the intensity of effort to improve HVR-related variables in shorter conditioning protocols [17, 38]. Additionally, some authors have reported that continuous and moderate-intensity exercise training has not caused noticeable beneficial changes in HVR [15,17].

In agreement with earlier studies [26,27], there were significant inverse relationships between prevailing HR and SDNN, RMSDD, and pNN50. The normalized standard HRV indexes were relatively attenuated since these variables had a reduction in the correlation degree concerning the average HR, except the Ln RMSSD that has become with a positive relationship between prevailing HR (r = 0.49; p <0.001). However, after the correction, the changes in Ln RMSSD were unclear for both groups. To the best of our knowledge, no study to date has evaluated the normalization of the Ln RMSSD in dogs, an HRV parameter log-transformed to reduce bias arising from the respiratory rate. Thus, the Ln RMSSD is not significantly influenced by breathing frequency, unlike other spectral indices of HRV and much higher reliability than other spectral indexes to monitor day-to-day the endurance training progress [32, 39]. Besides, studies previously reported in humans showed uncertain findings for Ln RMSSD [4]. Additionally, the pNN50 correction changed the behavior of this variable considerably, which is strongly influenced by respiratory rate in dogs, and further studies are needed to determine the normalization effect on the pNN50, notably when HR reduces induced by endurance training. In summary, some autonomic changes were not consistently related to adjustments in performance, and correcting by the HR may alter these relationships on trained dogs differently.

Exercise training induces beneficial cardiovascular response due to elevated HRV indices in physically fit individuals [16, 38, 39]. This benefit has also been reported in dogs with induced ventricular fibrillation [21, 40]. When viewed together, the trained dogs had increased SDNN, pNN50 (see before correction), RMSSD, and Ln-RMSSD; similar results have already been reported for humans submitted to different conditioning programs. The responses obtained herein may be explained by the increasing cardiac parasympathetic regulation, like findings described for humans. This similarity occurs due to the intrinsic characteristics of these two species, notably the cardiac neurological and electrophysiological properties [21, 41, 42].

The rise observed in the pNN50, and RMSSD measures in trained dogs suggested an increase in parasympathetic tone, making the training protocol used herein somehow effective and safe for dogs, since that the ETP increased the functional capacity inducing any ventricular or supraventricular arrhythmias. Scientific evidence has shown that endurance exercise could improve parasympathetic regulation by reducing sympathetic participation in healthy individuals [21]. Additionally, dogs with occlusion of the left circumflex coronary artery that underwent aerobic-training had risen parasympathetic regulation, and the authors stated that the aerobic protocol caused neither risk of atrial fibrillation nor myocardial fibrosis [21]. To ensure that the endurance-training protocol is prescribed in the correct intensity range so that it represents an oxidative contribution to ATP production, the LT method might be safely applied as a guide and become an exciting alternative for both, healthy and sick dogs.

In human athletes, studies have shown that using the Ln-RMSSD is more reliable to determine parasympathetic improvement in conditioned individuals, being an appropriate index to evaluate training evolution over time [43]. Our study, with a relative degree of novelty in dogs, showed an improved in Ln-RMSSD at weeks 6 and 8 of conditioning compared to the beginning of training. The advantage of using Ln-RMSSD to evaluate the effect of training on vagal response is related to the little influence of respiration on this variable since it is well known the possible occurrence of respiratory sinus arrhythmia in dogs and its importance when determining HR [44,45]. Thus, the present research showed that Ln-RMSSD could be considered a useful tool to determine parasympathetic action in dogs submitted to continuous exercise.

### Study limitations

Some limitations of our work need to be recognized. First, we could mention the lack of results for frequency-domain variables that might be complemented by the with the results for time-domain variables. This limitation could be overcome relatively easily as the HF Power, the frequency domain measure, is strongly correlated with time-domain measurements over 24-h such as pNN50 and RMSSD. Also, all time-domain variables influenced the group undergoing aerobic training. Second, the respiratory rate may change HRV. Therefore, changes induced by breathing conditioning, especially in expiratory muscles and pulmonary compliance [46], could have contributed to higher HRV after exercise training. Thus, in the subsequent studies on the relationship between exercise training and HRV in dogs, there is the necessity to study methods that investigate a possible improve the respiratory muscles submitted to an ETP.

In the present study, there was no a no-exercise control group submitted to two maximal incremental exercise tests (IET-1 and IET-2). We recognize that this would have caused the normal variability observed in the measurements from the IETs over time, as well as, environmental changes and treadmill protocol familiarity, and if the adjustments in VLT and Vmax were a result of the eight-week ETP. This condition could be a severe flaw in our experimental design. However, from our recent findings, a study with the same experimental procedure utilized herein [2] which showed that the positive control untrained group, who performed the same tests (IET-1 and IET-2) but did not undergo ETP, the VLT and Vmax did not differ between the IETs. VLT and Vmax increased in group T, with trained dogs reaching higher speeds in IET-2 than dogs of the control group. At the moment of evaluation VLT and Vmax were aproximadely 25% and 9% greater in the trained group than in control. Thus, it becomes clear that the changes found in this study, concerning VLT and Vmax for the trained dogs, were elicited by the training and not by familiarity with the treadmill. It is noteworthy that the control dogs had access to an outdoor playground for daily exercises for 4 hours. The objective was to avoid behavioral changes such as excess moving, as well as the exhibition of the significant number of bizarre movements, and vocalization [47].

In general, threshold-based training prescription attenuated the individual variation in VO_2_ max to aerobic exercise training in humans compared with a relative percent heart rate approach having a more significant impact of non-responders to endurance training [48]. However, due to the study design utilized herein, we do not affirm if the changes in HRV measures following ETP were a result of the prescription (i.e., lactate threshold-based) or as a consequence of endurance practice in general. As such, we are left with another study comparing the effects of regular submaximal aerobic exercise on heart rate variability.

Additionally, due to our logistics conditions, the control dogs were not exposed to the experimental environment as the noises associated with the treadmill and testing room. Stressful stimuli could elicit an effect on autonomic function [37]. Without these external factors of exposure to the kennel on the control dogs, it would be challenging to know the independent effects of the treadmill running. However, even being exposed to the stressful stimuli, our trained dogs revealed a reduction of the resting heart rate, and improvements were found in measures of parasympathetic tone (i.e., RMSSD and Ln-RMSSD) following endurance training.

### Clinical implications

CAR is adversely altered in many pathological conditions, including diabetes, kidney and heart diseases [11, 12, 49, 50]. Herein, we showed that the ETP has the potential to restore autonomic status, increasing parasympathetic tone or reduced sympathetic regulation. Thus, continuous aerobic fitness has beneficial effects on cardiovascular autonomic function. This response can be used in cardiovascular disorders as a cardioprotective. In this sense, the conditioning protocol based on 70 and 80% of the LT in dogs may become a non-pharmacological treatment, specifically cardiovascular diseases, preventing cardiac remodelling, improving welfare and increasing patient survival [51]. However, to this day, using exercise training for healthy in Veterinary Cardiology is still ignored [23, 52]. The findings of this study with healthy animals are essential for future studies, and the literature expresses the need for several controlled clinical studies using healthy patients to evaluate the results, efficacy, and safety of various conditioning modalities [53].

## Conclusions

The 8-week endurance-training program guided by the lactate-velocity curve increased the cardiac parasympathetic regulation, as shown by the increasing HRV and decreasing HR in dogs. This conditioning protocol can be applied to improve vagal activity in dogs. Furthermore, future studies are needed to determine whether this protocol has similar effects on dogs with diseases that alter vagal action.

## Supporting information

S1 Video (MP4)

S1 Fig (TIF)
