# Supplementary figures and images for: Effect of a lactate‐guided conditioning program on heart rate variability obtained using 24-Holter electrocardiography in Beagle dogs
Source: PLoS One. 2020 Jun 1;15(6):e0233264. doi: 10.1371/journal.pone.0233264 (PMC7263627; doi:10.1371/journal.pone.0233264)

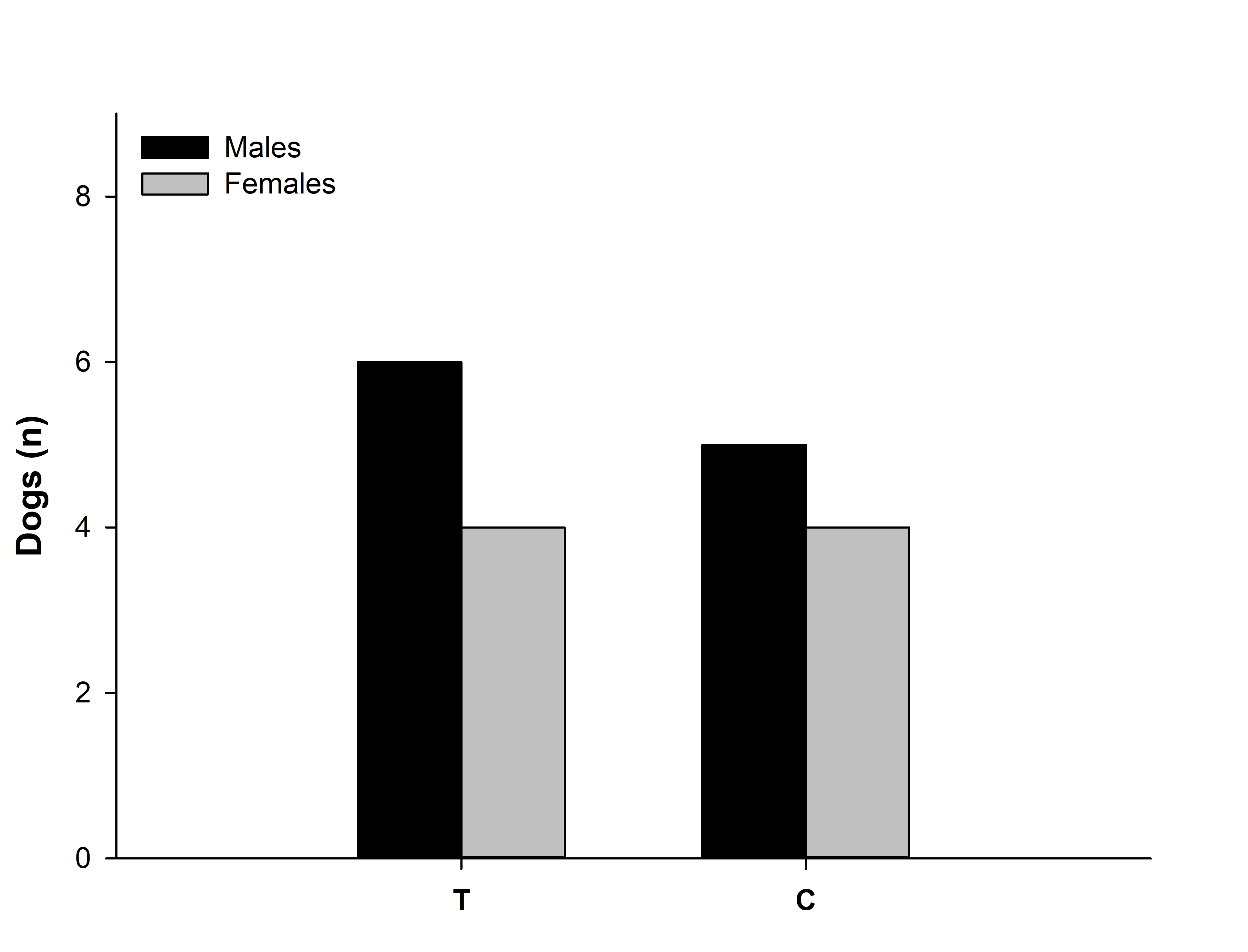

Supplement: S1 Fig — (TIF) [file pone.0233264.s002.TIF]
